# Aptamer-Based Biosensors for Environmental Monitoring

**DOI:** 10.3389/fchem.2020.00434

**Published:** 2020-05-29

**Authors:** Erin M. McConnell, Julie Nguyen, Yingfu Li

**Affiliations:** Department of Biochemistry and Biomedical Sciences, McMaster University, Hamilton, ON, Canada

**Keywords:** aptamers, biosensors, environmental monitoring, water quality, bacteria, heavy metals, environmental contaminants

## Abstract

Due to their relative synthetic and chemical simplicity compared to antibodies, aptamers afford enhanced stability and functionality for the detection of environmental contaminants and for use in environmental monitoring. Furthermore, nucleic acid aptamers can be selected for toxic targets which may prove difficult for antibody development. Of particular relevance, aptamers have been selected and used to develop biosensors for environmental contaminants such as heavy metals, small-molecule agricultural toxins, and water-borne bacterial pathogens. This review will focus on recent aptamer-based developments for the detection of diverse environmental contaminants. Within this domain, aptamers have been combined with other technologies to develop biosensors with various signal outputs. The goal of much of this work is to develop cost-effective, user-friendly detection methods that can complement or replace traditional environmental monitoring strategies. This review will highlight recent examples in this area. Additionally, with innovative developments such as wearable devices, sentinel materials, and lab-on-a-chip designs, there exists significant potential for the development of multifunctional aptamer-based biosensors for environmental monitoring. Examples of these technologies will also be highlighted. Finally, a critical perspective on the field, and thoughts on future research directions will be offered.

## Introduction

As the world population grows and the climate rapidly changes, the need for simple, cost-effective biosensors for environmental monitoring is becoming ever more apparent. Originally described by the World Health Organization as the ideal characteristics for point-of-care testing in low-resource settings, the ASSURED criteria stand as an excellent benchmark for the development of biosensors for environmental monitoring as well. These criteria state that in order to be practical for use, a biosensor should be affordable, sensitive, specific, user-friendly, rapid and robust, equipment-free, and deliverable (Weerathunge et al., 2019).

Though traditional methods boast high sensitivity and specificity, there are several challenges that remain in using these approaches for practical environmental monitoring. The necessity of expensive equipment and highly trained personnel notwithstanding, to assess samples using gold standard methods like mass spectrometry or HPLC, it is often required that samples are collected, transferred, and pretreated before analysis (Kudłak and Wieczerzak, 2020). Many environmental contaminants of interest could be degraded during these steps, further compounding the challenges of detecting target analytes which may already be at low concentrations in complex matrices. In an effort to minimize these chain of analysis errors, many researchers have made efforts to develop inexpensive, portable platforms, including but not limited to paper-based sensors and smartphone-based analysis, which can be used for on-site detection. Other researchers have developed highly sensitive and specific assays which can be adapted for use with commercially available hand-held units, such as UV-Visible and fluorescence spectrometers, and lab-on-a-chip type designs which utilize miniaturized field-effect transistors and electrochemical analysis platforms, for example.

In conjunction with these strategies, researchers are more commonly employing aptamers and nanomaterials as part of the solution to develop biosensors for environmental monitoring which move toward satisfying the ASSURED criteria. Aptamers are small, synthetically derived, single-stranded oligonucleotides which bind to their cognate target with high affinity and selectivity. They are identified through an iterative *in vitro* selection process, Systematic Evolution of Ligands by EXponential enrichment (SELEX), which can be tailored to produce molecules which are highly specific to one target analyte over potential interferents (Ellington and Szostak, 1990; Tuerk and Gold, 1990; Kudłak and Wieczerzak, 2020). Aptamers have been selected for targets ranging from small molecules to whole cells and bacteria (McKeague et al., 2015b). Aptamers can form diverse, complex secondary structures ranging from multi-branched loops or junctions, to G-quadruplexes, a property which is often exploited in the development of biosensors (Roxo et al., 2019; Sullivan et al., 2019). Aptamers are particularly well-suited for applications in environmental monitoring because they are chemically stable, easily chemically modified, relatively easy to synthesize, and biocompatible (Ruigrok et al., 2011). As such, researchers have previously been successful in using aptamers to build categorically diverse biosensors for the detection of a wide range of environmentally relevant analytes (Rapini and Marrazza, 2017; Cunha et al., 2018; Geleta et al., 2018; Mishra et al., 2018; Sun and Lu, 2018; Yan X. et al., 2018; Zhang et al., 2018e; Alkhamis et al., 2019; Moro et al., 2019; Verdian et al., 2019; Zhao Q. et al., 2019; Kudłak and Wieczerzak, 2020).

Combined with aptamers, nanomaterials add complexity and diversity to sensing systems, which allow for the design of stand-alone platforms which afford high sensitivity and specificity yet do not require the use of complex instrumentation or highly trained personnel. By definition, nanomaterials have at least one dimension that measures on the nanometer scale (<100 nm), often leading to relatively enhanced physical and chemical properties when compared to traditional materials. Nanomaterials, combined with the use of aptamers as the molecular recognition element, have been widely applied to develop optical, electrochemical, and mechanical sensors for environmental monitoring (Kaur and Shorie, 2019).

There still remains many challenges of aptamer-nanomaterial based sensors for environmental monitoring, including the incorporation of designed sensors into cost-effective, user-friendly, portable systems, and therefore many opportunities for researchers exist. This review focuses on highlighting examples where the described biosensors have either been incorporated into a portable sensing system, or have been developed such that their translation from the bench to on-site detection could potentially be facilitated by commercially available technologies. Specifically, aptamer-based biosensors for monitoring water, soil, and air are discussed. Further, the incorporation of aptamers into wearable and sentinel technologies are discussed in the context of opportunities for environmental monitoring.

## Aptamer-Based Biosensors for Monitoring Water Quality

The vast majority of aptamer-based biosensors for environmental monitoring detect targets with relevancy to water quality. Most commonly bacteria, bacterial toxins, or heavy metals were detected. Additional targets include aquatic toxins, pesticides, industrial byproducts, antibiotics, and pharmaceuticals. The following sections will highlight recent examples of biosensors developed for monitoring water quality.

### Aptamer-Based Biosensors for the Detection of Bacteria

The contamination of water sources by bacteria is an international problem resulting in both medical and economic burden. More than 2 million deaths per year are caused by water-borne diseases which are the direct result of contamination by pathogenic bacteria (Kumar et al., 2018). Contaminated drinking water, ground water, waste water, and other water sources can lead to wide-spread illness and death. Additionally, there is a complex interconnected relationship between contaminated water and contaminated soil which has profound impacts on the environment, human health, and the agricultural and aquacultural industries. Therefore, there is an immediate need to develop highly sensitive biosensors for the detection of water-borne bacteria. The following sections describe progress made toward the development of aptamer-based biosensors for the detection of *Salmonella, Escherichia coli, Staphylococcus aureus, Microcystis aeruginosa, Listeria monocytogenes, Pseudomonas aeruginosa, and Vibrio*. In many cases detection in real samples was demonstrated in beverages (or food) rather than water. Though these bacteria exist in water, and contaminated water may be a source of infection, there is oftentimes a greater practical interest in food monitoring. These examples were included though, as many other works have demonstrated that if detection in complex aqueous samples such as milk or juice is possible, detection in environmental water samples is likely possible.

#### Salmonella

The *Salmonella* species are gram-negative, flagellated anaerobic bacilli that are responsible for *Salmonella* infections, commonly referred to as salmonellosis. Depending on the specific strain of *Salmonella* an individual is infected with, an infection may clinically range from Salmonella gastroenteritis, which are characterized by diarrhea, and abdominal cramps, to enteric fevers (including typhoidal fever), which are an acute, life-threatening and febrile type of systemic disease that requires immediate antibiotic therapy. Salmonellosis is a major public health concern worldwide due to the numerous ways it can find its way into our digestive tracts, including contaminated water, in addition to the lack of adequate programs and devices to control *Salmonellae*.

Several different types of biosensors have been described for the detection of *Salmonella*. Zhang et al. recently developed an aptamer-based biosensor for the detection of *Salmonella enteritidis (S. enteritidis)* that incorporated AuNPs as a colorimetric reporter (Zhang Z. et al., 2019). Gold nanoparticles (AuNPs) exhibit a unique optical property, known as surface-enhanced plasmon resonance, which causes the absorbance peak of the AuNPs to shift from 520 to 620 nm (resulting in a change of color from red to blue/purple) in response to dispersion and aggregation. In addition to this, they are easy to modify with various ligands and are relatively stable under physiological conditions. Zhang et al. first performed a novel selection experiment and identified two high affinity aptamers with dissociation constants (*K*_d_) of 43.8 and 56.8 nM. These aptamers were incorporated into a AuNP system where, in the absence of the target bacteria, the aptamers adsorbed to the AuNP surface, and protected them from salt induced aggregation. When the target bacterium was present, the aptamer preferentially interacted with the target bacteria leaving the AuNPs susceptible to salt-induced aggregation. Using either of the identified aptamers, the biosensor was able to detect cells in the linear range of 10^4^-10^5^CFU/mL. Though detection of *Salmonella* in environmental water samples was not shown in this example, many similar AuNP-based systems were effective in detecting target analytes in environmental water samples.

**Figure 1:**
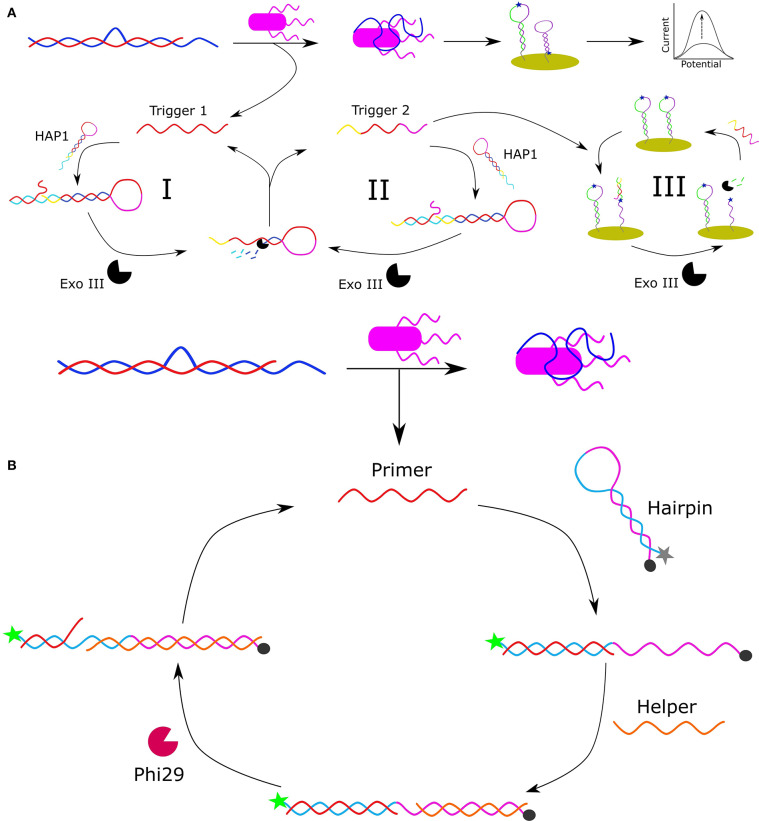
Schematic representation of apatmer-based electrochemical detection of *S. typhimurium* based on Exo III-assisted multiple signal amplification (Pei et al., 2019) **(A)**. Schematic representation of the FRET-based detection of *S. paratyphi* using a specific aptamer and Phi29-DNA polymerase-assisted cyclic signal amplification (Liang et al., 2019) **(B)**.

Another approach to detect *Salmonella* involved the design of an electrochemical biosensor, shown in Figure 1A (Pei et al., 2019). In this design, an arched probe, composed of a *Salmonella typhimurium* (*S. typhimurium*) aptamer (blue) and a complementary DNA segment (called the initial trigger DNA: red), was dissociated in the presence of *S. typhimurium* (purple). The aptamer used in this design was originally selected by Duan et al. (2013) and had an apparent K_d_ of 16.34 ± 0.45 nM. The released initial trigger DNA enters the first cycle of signal amplification (I) as it is bound to the 3′-protruding terminus of hairpin probe 1 (HAP1) to result in a blunt end to initiate Exo III-assisted multiple signal amplification. Exo III cleaves the trigger DNA-HAP1 structure to recycle the initial trigger DNA and form a secondary trigger DNA which enters the second round of signal amplification (II). In the second round of amplification, the secondary trigger DNA is recycled to reform HAP1 and generate more secondary trigger DNA. The reaction sample is then transferred to a gold electrode surface containing immobilized hairpin probe 2 (HAP2) via gold-thiol chemistry, which is comprised of a sequence complementary to the secondary trigger DNA that is labeled with the electroactive reporter methylene blue. The binding of the secondary trigger DNA and HAP2 results in a blunt end that initiates cleavage by Exo III to bring methylene blue within proximity of the gold electrode to allow an electrochemical signal to be detected. In the third amplification cycle, the secondary trigger DNA is recycled allowing for a greater electrical signal to be produced. This is the first time that Exo III-assisted autonomous multiple cycle amplification was used for signal-on electrochemical sensing of pathogenic bacteria, and Pei et al. observed a wide detection range of 1 × 10^1^ to 10^7^ CFU/mL with a very low detection limit of 8 CFU/mL. Additionally, they demonstrated the detection of their target bacteria in milk. By modifying the aptamer sequences, this platform has the potential to serve as a practical platform for the detection of numerous analytes to allow for simple and highly sensitive environmental and food safety monitoring.

Förster resonance energy transfer (FRET) is a mechanism by which energy is transferred from an electronically excited donor molecule to a ground-state acceptor molecule when the molecules are in close enough proximity. Common FRET pairs include complementary dyes, and fluorophore and quencher pairs (Ogawa et al., 2009). When the donor and acceptor are separated, the FRET quenching of the donor's fluorescence is abolished allowing for a fluorescent signal to be released. Based on this principle, Liang et al. developed a rapid and ultra-sensitive FRET-based aptasensor using Phi29-DNA polymerase-assisted signal amplification for the detection of *Salmonella paratyphi A* (Liang et al., 2019). The aptamer used in this work was originally selected by Yan et al. (2015), and had an evaluated affinity for *S. paratyphi A* of 27 ± 5 nM. In Liang et al.'s design (Figure 1B), an arched probe consisting of an aptamer specific for *S. paratyphi A* (blue) and a primer (red) is disassembled in the presence of *S. paratyphi A* (purple) as the aptamer is bound to the target resulting in the release of the primer sequence. The released primer binds to a free-floating hairpin probe resulting in the separation of the F-Q pair which consists of a 5′-carboxyfluorescein (6-FAM) fluorophore and a 3′-black hole quencher. The unbound portion of the linearized hairpin is then complementarily bound by a helper DNA sequence allowing Phi29-DNA polymerase to initiate the polymerase chain displacement reaction and generate a new primer sequence for further amplification of the fluorescent signal. The linear range under optimized conditions was 10^2^ to 10^8^ CFU/mL with a detection limit of 10^2^ CFU/mL. Liang et al. were also able to detect *S. paratyphi A* in a complex medium (milk) demonstrating its potential for environmental monitoring and food safety analysis.

#### Escherichia coli

*Escherichia coli* (*E. coli*) is a gram-negative, rod-shaped anaerobic bacterium that normally reside in the gastrointestinal tract of humans and animals, and is crucial for maintaining good health. However, there are also several pathogenic strains that have emerged as a result of acquiring virulence factors through plasmids, bacteriophages, transposons, and/or pathogenicity islands (Lim et al., 2010). *E. coli* infections from these pathogenic strains can result in enteritis, urinary tract infections, diarrhea, septicaemia as well as several other clinical infections including neonatal meningitis (Allocati et al., 2013). Regarding the mode of transmission, highly pathogenic strains of *E. coli* are commonly transmitted to humans via the consumption of contaminated food, such as raw or undercooked beef and milk. Other common modes of transmission includes the consumption of fecal contaminated water, fruits and vegetables, and other meat products.

**Figure 2:**
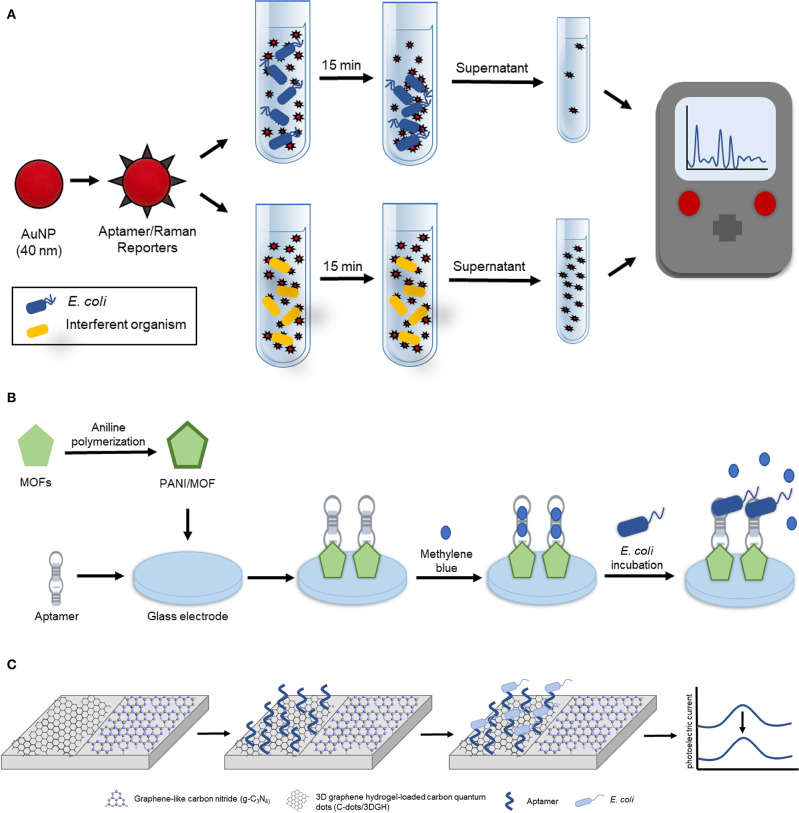
Aptamer-based detection strategies for *E. coli*. **(A)** Schematic illustration of the aptamer-based SERS biosensor for whole cell detection of *E. coli* O157:H7 (Díaz-Amaya et al., 2019). **(B)** Schematic representation of electrochemical aptasensor fabrication based on amino-functionalized metal-organic frameworks and *E. coli* O157:H7 detection (Shahrokhian and Ranjbar, 2018). **(C)** Schematic diagram of the ratiometric photoelectrochemical aptasensor for the detection of *E. coli* (Hua et al., 2018).

Recently, Stanciu and colleagues developed a surface-enhanced Raman spectroscopy (SERS) based aptasensor for the detection of *E. coli* O157:H7 capable of detecting and quantifying ~10^1^ CFU/mL in pure culture and 10^2^ CFU/mL in ground beef samples (Díaz-Amaya et al., 2019). In their design (Figure 2A), thiolated aptamers specific for *E. coli* O157:H7 were conjugated to the 4-aminothiophenol-AuNPs exploiting gold-thiol interaction and the resulting aptamer/Raman reporters were used to selectively bind and coat *E. coli* O157:H7. Following a 15-min incubation period, the aptamer/Raman reporter-bacteria complexes gradually precipitated, resulting in the formation of bacterial sedimentation. This sedimentation was visible by the naked eye at high concentrations of bacteria (10^5^ and 10^6^ CFU/mL). In order to quantify and detect low concentrations of *E. coli* O157:H7, the supernatant was removed, and the SERS signal was analyzed. Increasing concentrations of *E. coli* O157:H7 corresponded with a decreased SERS signal. In addition to this, Stanciu and colleagues compared their aptasensor to current immune-capture detection methods and found that although their method had a slightly higher limit of detection (LOD), it provided the advantage of being able to detect bacteria in a complex medium. For these reasons, this aptasensor has the potential to serve as a unique, highly sensitive and low-cost tool for the detection of a wide variety of pathogenic bacteria.

The following subsection highlights two electrochemical biosensors for the detection of *E. coli*. The first highly sensitive electrochemical biosensor was recently developed by Zhang et al. for the detection of gram-negative bacteria using commercially available microelectrodes functionalized with a liposaccharide (LPS) specific thiolated aptamer (K_d_ = 12 nM, Kim et al., 2012), and *E. coli* as the model gram-negative bacteria to develop and optimize the biosensor (Zhang et al., 2018d). This assay could detect as few as 267 cells/mL in as little as 30 s, however detection in an environmental sample was not demonstrated. The second electrochemical biosensor was described by Shahrokhian and Ranjibar. In this study Differential Pulse Voltammetry (DPV) was utilized to develop a novel aptamer-based diagnostic platform for *E. coli* O157:H7 using amino-functionalized metal-organic frameworks (MOFs) (Shahrokhian and Ranjbar, 2018). In their design (Figure 2B), the amino-functionalized-metal-organic frameworks (MOFs), whose electrical and morphological properties were enhanced with polyaniline (PANI), served as a nanocomposite to enhance aptamer immobilization onto the glass electrode. This enhancement was due to the PANI/MOF nanocomposite that significantly increased the surface area available for aptamer immobilization as the amino-modified *E. coli* O157:H7 specific aptamer (Wu et al., 2012) was covalently attached using glutaraldehyde as a crosslinking agent. To achieve electrochemical detection of *E. coli* O157:H7, the resulting biosensor was incubated in a solution containing methylene blue which adsorbed onto the aptamer. The DPV signal of the methylene blue intercalated biosensor was measured to obtain the reference DPV signal indicative of the absence of *E. coli* O157:H7. In the presence of *E. coli* O157:H7, the aptamer was bound to the bacteria resulting in the release of methylene blue, and a subsequent detectable change in the DPV signal. This platform design had a detection range of 2.1 × 10^1^ to 2.1 × 10^7^ CFU/mL and demonstrated the ability to accurately detect *E. coli* O157:H7 in real samples as Shahrokhian and Ranjibar tested their device in tap, mineral, well, and paddy water spiked with 2.1 × 10^3^ CFU/mL of *E. coli* O157:H7 demonstrating a recovery range of 73.2–113.6%.

Finally, a photoelectrochemical strategy for the detection of *E. coli* in milk was described. In this study, Hua et al. developed a reliable and highly sensitive potentiometric resolved ratiometric photoelectrochemical aptasensor for the detection of *E. coli* with a linear range of 2.9 CFU/mL to 2.9 × 10^6^ CFU/mL and a LOD of 0.66 CFU/mL (Hua et al., 2018). In their design (Figure 2C), three-dimensional graphene hydrogel-loaded carbon quantum dots (C-dots/3DGH) and graphene-like carbon nitride (g-C_3_N_4_), two non-metallic photoelectrochemical nanomaterials with excellent photoelectrochemical activity and matched potential, were placed on adjacent sides of an indium tin oxide (ITO) glass electrode. When the voltage was set to 0.15 V, which is the critical voltage of g-C_3_N_4_ under light irradiation, the resulting cathodic current from the g-C_3_N_4_ was zero causing the photoelectric current of the ITO electrode to be entirely generated by the C-dots/3DGH anodic current. Similarly, when the bias voltage is switched to −0.4 V, the critical voltage of the C-dots/3DGH, the photoelectric current of the ITO glass electrode is entirely the result of the g-C_3_N_4_. To allow for *E. coli* detection, an *E. coli* specific amino-modified aptamer (K_d_ = 25.2 nM, Kim et al., 2013) was immobilized onto the surface of the C-dots/3DGH to result in the capture of the pathogen to the sensor when it is present in solution. When the bias current is set at 0.15 V, the immobilization of *E. coli* introduces steric hindrance to alter the C-dots/3DGH cathodic current resulting in a detectable decrease in the photoelectric current. Meanwhile, the anodic g-C_3_N_4_ current is used as a stable reference to account for other analytes in the solution that may alter the photoelectric current. In addition, the concentration of *E. coli* was also able to be quantified using the ratiometric difference between the anodic and cathodic currents. The performance of the sensor was further tested in milk samples spiked with *E. coli* and the recovery rate was determined to be within the range of 98.6–102.0%.

#### Staphylococcus aureus

The detection of *Staphylococcus aureus* (*S. aureus*) is of particular importance because of its ability to cause diverse infections ranging from food poisoning to pneumonia (Cai et al., 2019). Additionally, *S. aureus* can contaminate air, water, and soil and therefore is relevant as an analyte to food safety and environmental monitoring alike.

Toward the goal of achieving simple, portable detection of *S. aureus*, Shrivastava et al. developed a fluorescent aptasensor using *S. aureus* specific carboxylated aptamer (K_d_ = 35 nM, Chang et al., 2013) functionalized to amine-modified fluorescent magnetic nanoparticles (FMNPs), a bacterial capture chip and a smart phone to detect the fluorescent signal (Shrivastava and Lee, 2018). This method could detect as low as 10 CFU/mL in peanut milk samples within 10 min. The authors suggest that this method could allow for on-site detection of bacteria especially in remote and resource-limited settings. Oftentimes the detection of *S. aureus* is demonstrated in food or beverage matrices rather than water, as in the case with this example. However, this particular example was highlighted as it reported a simple, portable device with very high sensitivity and rapid assay time which could certainly be translated to the detection of bacteria in environmental water samples.

**Figure 3:**
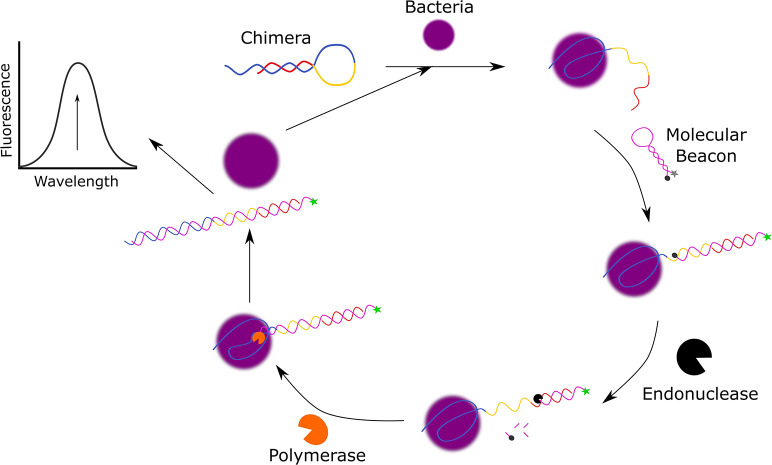
Schematic diagram of the fluorescent detection of *S. aureus* using molecular beacon-controlled signal amplification (Cai et al., 2019).

One example in which detection of *S. aureus* was demonstrated in environmental water samples was reported by Cai et al. In this design (Figure 3), the functional chimera sequence used for the ultrasensitive detection of *S. aureus* was made up of a *S. aureus*-specific aptamer sequence (K_d_ = 3.49 ± 1.43 nM, Moon et al., 2015), a Bpu10I endonuclease recognition sequence in the middle and a partially complementary sequence to the aptamer sequence to allow for the formation of a hairpin structure (Cai et al., 2019).

The molecular beacon (MB) also takes on the form of a hairpin and is used as a signal transducer, as it contains a 5′-FAM fluorophore and 3′-4-(dimethylaminoazo)-benzene-4-carboxylic acid (DABCYL) quencher which are only within close proximity in the absence of *S. aureus*. In the presence of *S. aureus*, the *S. aureus* specific aptamer sequence of the chimera structure is bound to the bacteria resulting in the unfolding of the hairpin structure as the 3′-terminus is released. The free 3′-terminus of the chimera induces the unfolding of the MB to hybridize with the 3′-terminus of the chimera and emit a fluorescent signal. To further amplify this fluorescent signal, NbBpu10I endonuclease and Bsm DNA polymerase are added to the system to cleave the hybridized MB resulting in the release of the 3′-FAM and to displace *S. aureus* from the complex to allow it to initiate another cycle of reactions. This assay has a broad linear range from 80 CFU/mL to 8.0 × 10^6^ CFU/mL, a LOD of 39 CFU/mL, and the ability to discriminate against bacteria. In addition to this, Cai et al. have also demonstrated that this detection method can also function in complex media as they successfully detected spiked *S. aureus* in local tap water, lake water from Taihu lake, and pond water from the local pond with recoveries that ranged from 97.1 to 104.2%.

#### Microcystis aeruginosa

**Figure 4:**
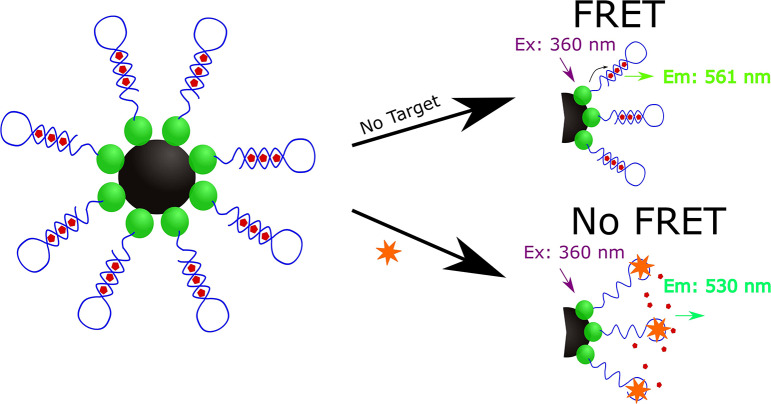
Schematic illustration of the quantum dot mediated FRET based indirect detection of *M. aeruginosa* based on the direct detection of microcystin-LR (Lee and Son, 2019).

Lee and Son designed a FRET-based aptasensor for the indirect detection of *Microcystis aeruginosa* by the direct detection of microcystin-LR (MC-LR), a toxin that has been demonstrated to cause liver damage and may even stimulate the growth of cancer cells in response to short and long-term exposure, respectively (Lee and Son, 2019). Additional MC-LR examples are described in the aquatic toxin section. To develop this aptasensor (Figure 4), carboxylic acid-modified quantum dots (QD_525_) were immobilized onto aminated magnetic beads (MagB) and the QD_525_-magnetic bead conjugates were then covalently bound to amine-modified aptamers specific for MC-LR (K_d_ = 50 ± 12 nM, Chang et al., 2013).

The resulting complex (MagB-QD_525_-aptamer) was incubated with the PoPo^TM^-3 iodide (PoPo3) dye to allow the dye to intercalate within the aptamer sequence of the MagB-QD_525_-aptamer structure. At 360 nm, the QD_525_ nanoparticles are excited and act as a donor fluorophore as it emits a 530 nm wavelength to excite the accepter PoPo3 dye to result in the emission of a 561 nm wavelength. In the presence of MC-LR, only the 360 and 530 nm wavelengths are detected as the binding of MC-LR to the aptamer displaces PoPo3 dye to result in the absence of the 561 nm signal due to long-range dipole-dipole coupling when analyzed with a spectrofluorometer. To assess the selectivity of the sensor, Lee and Son used other variants of microcystin (microcystin-YR, microcystin-LY, microcystin-LW, microcystin-RR, microcystin-LF, microcystin-LA, and Nodularin) and found that the sensor demonstrated significantly higher specificity for MC-LR. Lee and Son also demonstrated that this aptasensor could detect MC-LR in eutrophic water samples collected from the Han River in South Korea spiked with *M. aeruginosa*.

#### Listeria monocytogenes

*Listeria monocytogenes* is a food-borne bacterium that can also contaminate water. Suh et al. sought to develop an aptamer-based biosensor for the detection of *L. monocytogenes* in food and environmental samples (Suh et al., 2018). In their two-site binding sandwich assay, the first binding of *L. monocytogenes* was achieved by using biotinylated anti-*L. monocytogenes* antibodies that had been bound to streptavidin-coated magnetic beads for the capture of *L. monocytogenes*. The second binding of the pathogen was achieved by adding LM6-116 (a 5′-FAM-labeled aptamer specific for *L. monocytogenes* with a K_d_ of 74.4 ± 52.7 nM, Suh et al., 2014) to the solution to result in the two-site binding sandwich structure as *L. monocytogenes* was bound by both the antibody and aptamer. After a 45-min incubation period, the washed samples were placed in a 90°C hot water bath to release and denature LM6-116, which was then collected and used as a template for RT-qPCR. Using this method, as low as 2.5 CFU of *L. monocytogenes* in 500 μL of buffer was achieved. This assay was also applied to detect the bacteria in turkey deli meat at a level of 1–2 log_10_ CFU per 25 g of meat.

#### Pseudomonas aeruginosa

*Pseudomonas aeruginosa* is a bacteria that is ubiquitous in the environment. In the proposed *P. aeruginosa* detection method, Shi et al. developed an electrochemical biosensor with a detection limit of 9 CFU/mL in buffer and 52 CFU/mL in spiked blood samples (Shi et al., 2019). In their design, a 5′-biotin-labeled aptamer specific for *P. aeruginosa* (K_d_ = 17.27 ± 5.00 nM, Wang K.-Y. et al., 2011) was immobilized onto streptavidin-coated magnetic beads which were then hybridized to a poly(A)-detection probe to prepare the MagB-aptamer-poly(A)DNA. In the presence of *P. aeruginosa*, the MagB-aptamer-poly(A)DNA was bound to the pathogen, resulting in the release of the poly(A)-detection probe as the result of the strong interaction between the *P. aeruginosa*-specific aptamer. The released poly(A)-detection probe was then adsorbed onto the gold interdigital electrode surface due to the high affinity between the adenine bases of the probe and gold. This adsorption was detected as a signal shift in the frequency of the multi-channel series piezoelectric quartz crystal (MSPQS) sensor connected to the electrode. This biosensor also proved to be highly specific as the authors tested the method using 1 × 10^3^ CFU/mL of *E. coli, S. aureus, Enterococcus faecalis, Klebsiella pneumoniae, Streptococcus pyogenes, and Enterobacter cloacae* and demonstrated that the biosensor selectively detected *P. aeruginosa*. The proposed biosensor serves as a novel, simple, rapid and highly sensitive detection of *P. aeruginosa* in the food, clinical, and environmental sectors.

#### *Vibrio* Species

The *Vibrio* species, gram-negative anaerobic bacilli that commonly reside in aquatic environments including fresh, estuarine, and surface water of varying salinity and temperatures, are of interest for the development of biosensors for environmental monitoring (Osunla and Okoh, 2017). Of the *Vibrio* species, some of the most researched and documented are *Vibrio parahaemolyticus, Vibrio cholerae, and Vibrio vulnificus* due to their pathogenicity against human health. For these pathogenic strains, the main mode of transmission usually involves the consumption of undercooked seafood harboring the pathogen or contaminated water. Aptamers were recently selected for *Vibrio vulnificus* and *Vibrio alginolyticus*, the latter of which was assembled into a biosensor that took advantage of asymmetric PCR to detect as few as 8 CFU/mL (Yan W. et al., 2018; Yu et al., 2019).

#### Portable and Multiplex Detection of Bacteria in Water

**Figure 5:**
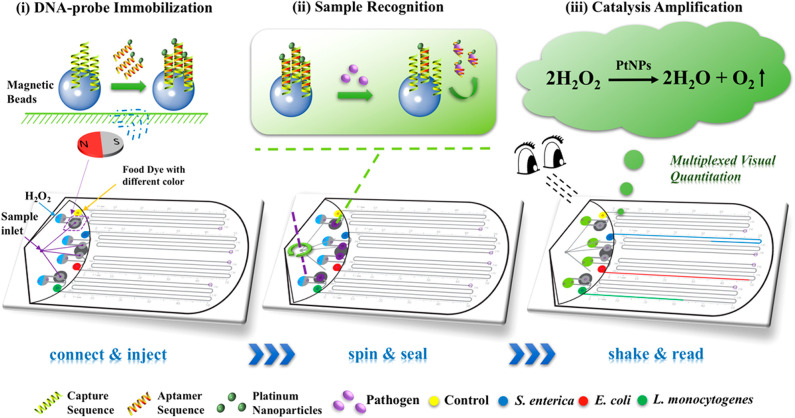
Schematic diagram of the multiplexed instrument-free bar-chart SpinChip integrated with nanoparticle mediated magnetic aptasensors for quantitative visual detection of multiple pathogens including *S. enterica, E. coli*, and *L. monocytogenes*. Reprinted (adapted) with permission from Wei X. et al. (2018). Copyright (2018) American Chemical Society. Permission has been obtained from American Chemical Society.

Wei et al. developed a portable multiplex bar-chart SpinChip (MBC-SpinChip) incorporating aptamer-specific recognition and magnetic nanoparticle-catalyzed pressure amplification for the simultaneous visual quantitative detection of multiple pathogens (Wei X. et al., 2018). In this paper, the MBC-SpinChip (Figure 5) demonstrated the ability to detect *S. enterica, E. coli, and L. monocytogenes*, three major foodborne pathogens, in apple juice with limits of detections of ~10 CFU/mL. The MBC-SpinChip is comprised of four components: the spin unit, sample recognition unit, catalytic amplification unit, and bar-chart unit. The MBC-SpinChip apparatus begins with the spin unit which is where samples are injected. By continuously rotating the spin unit following sample injection, the reaction sample is equally distributed into four parallel channels that lead to four different sample recognition units. In these units, there are DNA probes formed by DNA hybridization between a magnetic bead-capture DNA and a platinum nanoparticle (PtNP)-*S. enterica/E. coli/L. monocytogenes* specific aptamer. These DNA probes are pre-immobilized onto the sample recognition unit of the apparatus via a magnetic field. In the presence of the pathogen, the PtNP-aptamer is released from the DNA probe to form free-floating complexes. After the recognition reaction, the resulting samples are shaken down into the catalytic amplification unit which contains H_2_O_2_. Oxygen gas (O_2_) and water are rapidly generated as the PtNP of the free-floating PtNP-aptamer catalyzes the reaction resulting in a dramatic increase in pressure in the sealed chamber. As the pressure built up, food dye within the bar-chart unit was pushed along the bar-chart channels allowing for visual detection of the pathogen. In addition to this, because pathogen concentration is proportional to the moving distance of food dye, pathogen quantification was also possible using the MBC-SpinChip. This simple-to-use, instrument-free, and user-friendly device that has great potential for the detection of a wide range of pathogens for food safety, environmental surveillance, and diagnosis of infections.

### Aptamer-Based Biosensors for the Detection of Heavy Metals in Water

The contamination of water sources by heavy metals, such as arsenic, cadmium, lead, silver, and mercury, can lead to multiple adverse effects on human health and aquatic life (Yadav et al., 2019). These issues are further compounded by the tendency of heavy metals to bioaccumulate in living systems (Malik et al., 2019). Liquid effluent and industrial waste are major contamination sources to natural water systems like river and groundwater (Yadav et al., 2019). One of the most common strategies for heavy metal detection is to employ electrochemical methods (Rapini and Marrazza, 2017; Mishra et al., 2018; Malik et al., 2019). However, optical and mechanical methods have also been described (Khoshbin et al., 2018; Zhang et al., 2018e; Kudłak and Wieczerzak, 2020).

#### Arsenic

Several aptamer-based biosensors for the detection of arsenic have been described which utilize an aptamer first reported by Kim et al. (2009) and Mao et al. (2020). These biosensors utilized optical and electrochemical methods which demonstrated limits of detection in the nM range, with some notable exceptions reaching limits on the order of magnitudes of 10^−3^ and 10^−6^ nM. The following examples highlight the use of aptamer-based biosensors for the detection of arsenic in environmental sources.

Recently, Yadav et al. reported an electrochemical biosensor which employed glassy carbon electrode modified with aptamer-coated Ag-Au alloy nanoparticles (Yadav et al., 2019). The biosensor demonstrated detection of As^3+^ in the linear dynamic range of 0.01–10 μg/L and a detection limit of 0.003 × 10^−3^ μg/L. These specifications are suited well to the regulatory limit of 10 μg/L recommended by the World Health Organization (Ahmad and Bhattacharya, 2019). Additionally, the biosensor was used to detect As^3+^ obtained from six different ground water sources in India as well as one river sample. This versatile design was assessed using cyclic voltammetry and differential pulse voltammetry. Spiked recoveries ranged from 92 to 108% and measured concentrations of As^3+^ present in these water sources ranged from 190 to 393 μg/L.

Employing a simple fluorescent strategy, Ravikumar et al. developed an aptamer-based “turn-on” biosensor for the detection of arsenic in environmental water samples. In this design, an arsenic binding aptamer was terminally labeled with a fluorophore (FAM) (Ravikumar et al., 2018). In the absence of arsenic, the aptamer interacted with MoS_2_ sheets, which quenched the fluorescence. In the presence of arsenic, the metal interacted with the aptamer via coordination through the phosphate and amine groups of the DNA, ultimately leading to the desorption of the aptamer from the surface of the MoS_2_ sheet, and the consequent, concentration-dependent increase in fluorescence signal. Rapid and selective detection of arsenic was achieved in ~30 min from preparation to fluorescence measurement. A selective signal was generated in the presence of As (III) compared to common ions Cu (II), Ni (II), Mn (II), Zn (II), Cd (II), Pd (II), Hg (II), Co (II), Ba (II), Fe (II), and AS (V). This assay had a reported LOD of 18 nM. Finally, the biosensor demonstrated recoveries ranging from 95.6 to 101.8% when tap water and lake water were spiked with 5, 10, or 20 nM of As (III).

A distinct advantage of aptamers, and other functional nucleic acid-based approaches for biosensor design is the potential to incorporate known nucleic acid-based signal amplification strategies. Pan et al. reported the design of an ultrasensitive aptamer-based biosensor for the detection of arsenic in environmental water samples which utilized a triple-helix molecular switch, enzyme-based signal amplification, and fluorescence (Pan et al., 2018). Specifically, a hairpin structure (named THMS) was designed which included an aptamer region and a signal transduction probe (STP) binding region. An additional hairpin (HP1) was designed to contain a C-C mismatch for selective binding of the fluorescent reporter molecule (ATMND: 2-amino-5,6,7-trimethyl-1,8-naphthyridine) and regions of complementarity to STP and a third DNA probe (secondary STP). When no arsenic was present, ATMND interacted with the cytosine mismatch in HP1, and the fluorescence was quenched. When As (III) was present in solution, the aptamer region of THMS preferentially bound to the As (III), releasing the STP, which then interacted with HP1 by binding to the terminal complementary regions. Exo III assisted digestion of the terminal duplex region of HP1 formed by the STP binding released both STP and ATMND. Now free in solution, the released ATMND exhibited a strong fluorescence signal. This cyclic process allowed for signal amplification. Further amplification was achieved due to the presence of the secondary STP, which was generated as a digestion product of the first cyclic reaction, and can bind to the HP1/ATMND complex triggering Exo III digestion and ATMND release. A linear dynamic range of 10 ng/L to 10 mg/L, with a LOD of 5 ng/L, was observed. Further, the selectivity of the biosensor against common interfering ions (As^5+^, Ag^+^, Cd^2+^, Cu^2+^, Mg^2+^, Mn^2+^, Ni^2+^, Pb^2+^, and Zn^2+^) was demonstrated. The performance of the biosensor in the analysis of environmental water samples was evaluated by measuring the recovery of spiked ground water, and by measuring the actual As^3+^ concentration in tap, lake, and pond water as compared to LC-MS/MS controls. Recoveries of spiked ground water ranged from 97 to 110.5%, and the relative error between the proposed method and LC-MS/MS controls ranged from −5.25 to 3.90%.

To conclude this section, it is important to note that a handful of aptamers have garnered attention, and debate from researchers in the field due to their inconsistencies in binding, with the arsenic aptamer being one such example. Though several independent research groups have reported aptamer-based biosensors for the sensitive and selective detection of arsenic, Liu and colleagues recently reported that the commonly used arsenic aptamer Ars-3 reported by Kim et al. (2009) does not bind arsenic (Kim et al., 2009; Zong and Liu, 2019). In general, a challenge of aptamers is that they do not always consistently perform in one assay to another, highlighting the necessity of thorough characterization prior to biosensor development (McKeague et al., 2015a). In the particular case of the arsenic aptamer, many of the previously reported biosensors utilized AuNPs. Liu and colleagues recently presented evidence that in fact arsenic, in the form of arsenite [As (III)], adsorbs to the surface of the particle directly non-specifically displacing DNA, rather than through some specific interaction with the aptamer (Zong et al., 2019). Similar mechanisms may also explain why in many cases, though the original selection paper reported similar affinity to As (III) and As (V), many biosensors report specific affinity to As (III) (Zong and Liu, 2019). Further, these studies illustrate the need for extensive evaluation of controls in aptamer-based biosensors, for example, the biosensor platform's response to the target analyte should always be evaluated in the absence of the specific aptamer, and appropriate non-binding oligonucleotide controls should also be utilized. It is important for researchers to keep these examples in mind while designing and characterizing aptamer-based biosensors so as to optimize the performance and reliability of future assays.

#### Cadmium

**Figure 6:**
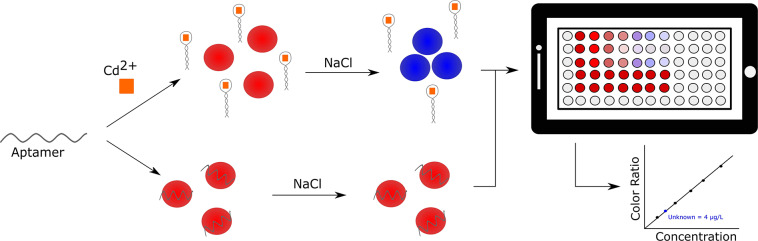
Smartphone-based detection of cadmium in environmental water samples. Specific detection is achieved using a gold nanoparticle assay (left side of panel) and the generated signal is processed and analyzed by a smartphone application (right side of panel) (Gan et al., 2020).

Another heavy metal of interest is cadmium. Like other heavy metals, it is often released into the environment via industrial and domestic wastewater, and can bioaccumulate to the detriment of human and wildlife health. Therefore, there is an urgent need for rapid and cost-effective detection of cadmium in environmental samples. In an effort to develop a relatively inexpensive and high throughput method for heavy metal detection, Gan et al. described the use of an aptamer and AuNP based sensor by which Cd^2+^ could be detected within 10 min using a self-developed smart phone-based colorimetric system (SBCS; Figure 6) (Gan et al., 2020). The cadmium binding aptamer used in this work was originally selected by Wu et al. (2014) and had a demonstrated dissociation constant of 34.5 nM. This assay makes use of the typical aptamer-AuNP design, where the AuNPs are protected from NaCl induced aggregation by adsorbed aptamer in the absence of Cd^2+^ ions, however it incorporates a smartphone containment unit and microplate loading station. This allows for the rapid quantitative determination of Cd^2+^. The sensor showed selectivity toward Cd^2+^ when assessed against common interfering ions (Pb^2+^, K^+^, Cu^2+^, Ca^2+^, Mg^2+^, and Na^+^). The sensor demonstrated a linear dynamic range of 2–20 μg/L and a LOD of 1.12 μg/L. Compared to similar AuNP based methods, this assay had the second lowest LOD, but had the added advantages of portability and high throughput processing. Further, Cd^2+^ concentration recoveries of 108.2–117.8% from spiked tap water were observed using the SBCS. Given the common use of the fundamental sensing component of the SBCS it is likely this system could be easily adapted to detect not only other heavy metals, but also other environmentally relevant targets (Chang et al., 2019).

#### Lead

One of the most common heavy metal pollutants is lead. Due to its physical properties and common use in things like batteries and gasoline, lead accumulates, and contaminates both ground water sources and soil (Yang et al., 2017). There is a particular need to develop highly sensitive and portable devices to detect lead due to its toxicity at relatively low levels. For reference, the United States Environmental Protection Agency (EPA) has defined the maximum contamination level as 72 nM (and 15 ppb or 15 μg/L) (Yang et al., 2017). Traditional methods, such as atomic adsorption spectrometry and mass spectrometry, for lead detection require costly equipment and highly trained personnel.

Yang et al. recently reviewed the progress made in the development of aptamer-based biosensors for the detection of lead (II) (Yang et al., 2017). Several optical and electrochemical strategies were reported, all having their own advantages. Fluorescence and colorimetric strategies offer simplicity, cost effectiveness and portability, whereas electrochemical methods are highly sensitive and can be miniaturized and incorporated into portable sensing platforms. One of the challenges that still faces aptamer-based lead detection is the lack of translation from bench to product, largely due to the challenges associated with lead detection in complex environmental samples (Yang et al., 2017).

Work done by Zhang and Wei explored the development of a turn-on fluorescent biosensor which exploited lead ion-dependent conformational change of a G-rich sequence to produce a fluorescent signal from silver nanoclusters (Zhang and Wei, 2018). In this design, the oligonucleotide sequence contained an aptamer region that was flanked by terminal Ag nanocluster nucleation sites. In the presence of lead (II), the aptamer underwent a conformational change which brought the Ag nanoclusters into close proximity of each other, thereby enhancing their fluorescence, and generating a concentration-dependent turn-on response. The sensor was able to selectively and reliably detect lead (II) between the concentrations of 5–50 nM, with a detection limit of 3 nM. The recovery of spiked lake and tap water was demonstrated between 96.90 and 105.5%.

The aptamer, PS2.M, used in this work has an interesting story. It was not initially selected for the target for which it is being used in this assay. In fact, PS2.M started as a DNAzyme rather than an aptamer, and remains one of the most commonly employed peroxidase mimic DNAzymes in biosensor design (Cheng et al., 2009). Originally Sen and colleagues selected the parent sequence to PS2.M as an aptamer to N-methylmesoporphyrin, then investigated it for its ability to act as a catalyst for porphyrin metalation, before in turn discovering that it could catalyze peroxidation reactions – seminal work in the field of catalytic functional nucleic acids (Li et al., 1996; Li and Sen, 1997; Travascio et al., 1998, 1999, 2001). Finally, Zhang and Wei were inspired by work done which demonstrated that other G-rich aptamers, such as the thrombin aptamer, could be conformationally controlled in the presence of lead (Lin et al., 2011), which led them to investigate the PS2.M sequence.

#### Silver

Silver is used extensively in a wide range of products from jewelry to medical technologies to gym socks, which has led to its environmental contamination and bioaccumulation. In small doses, silver can be a powerful antimicrobial agent, but this highly bioactive metal ion can cause serious health effects in humans. Like the detection of other metal ions, silver detection is usually achieved by atomic adsorption or emission spectroscopy and mass spectrometry, which face the same challenges (ease of use, portability, etc) for practical environmental monitoring. Taking advantage of non-canonical interactions of DNA with Ag ion, a molecular switch biosensor was designed to detect Ag^+^ in water samples (Zhang and Wang, 2019). Like the mercury aptamer, this silver ion binding probe was rationally designed to exploit G-Ag^+^-G interactions. The fundamental principle of the design relies on the differing rotational properties between the silver-bound and non-bound aptamer states. By measuring the fluorescence anisotropy, a value determined from the conformationally dependent interaction of sequence guanines and the fluorophore tetramethylrhodamine, concentration dependent fluorescent signal was generated within the dynamic concentration range of 2.0–100 nM with a detection limit of 0.5 nM. The selectivity of the probe was demonstrated in the presence of 14 common interfering metal ions. Recovery of Ag^+^ from spiked drinking (97.6 and 98.8%) and river (71.8 and 83.6%) water was also demonstrated.

#### Mercury

The history of the mercury binding aptamer and its use in biosensing has been well-documented (Zhou et al., 2017). The mercury binding aptamer is another example of a rationally designed sequence based on non-canonical DNA interactions. In this case, a T-Hg^2+^-T interaction is formed to stabilize a molecular beacon, or initiate some other sensing mechanism. Since mercury is a highly toxic metal which bioaccumulates, especially in aquatic systems, it is necessary to develop rapid, simple, and cost-effective sensing devices. To this end, several groups have described the development of aptamer-based biosensors for the detection of Hg^2+^ in water.

One approach utilized aptamer-modified SiO_2_@Au core/shell nanoparticles for the detection of Hg^2+^ in well and lake water at mid-micromolar concentrations (Lu et al., 2018). The aptamer was modified with an HS-(CH_2_)_6_- for conjugation to the surface of the nanoparticles. This label-free SERS approach allowed for the detection of Hg^2+^ over the concentration range of 10 nM−1 mM, while detecting as low as 10 nM. This method compared well with other strategies that utilized the T-rich DNA aptamer, but had the advantage that no additional Raman labeling was required to generate a SERS signal. Specifically, the authors took advantage of the intrinsic Raman signals of the purine bases to detect the interaction of Hg^2+^ with thymine. By this mechanism a Hg^2+^ induced structural change led to a measurable, concentration-dependent signal.

A common strategy for developing potentially portable biosensors is the use of fluorescence as a reporting signal, given that hand-held fluorescence spectrophotometers are commercially available. A relatively simple turn-off biosensor was reported in which detection of Hg^2+^ was dependent on the physical separation via magnetic beads of immobilized fluorescent reporter probe (when no Hg^2+^ was present) and fluorescent reporter probe that was free in solution (when Hg^2+^ present) (Sun et al., 2018). Magnetic nanoparticles were prepared through the immobilization of biotinylated aptamer to surface streptavidin. Using this biosensor, the authors reported a concentration-dependent signal increase from 2 to 160 nM, with a LOD of 0.2 nM. Additionally, the method was also used to quantify spiked Hg^2+^ in samples from river water and ribbon fish.

A distinct advantage of rationally designed oligonucleotide sequences with aptamer-like properties, such as the Hg^2+^ binding aptamer, is that their binding and interactions are understood well enough to combine them with computational approaches to biosensor design. In fact, sequences which exploit either the T-Hg^2+^-T or C-Ag^+^-C interactions have been used in DNA computing applications (Nie et al., 2018). Recently, Nie et al. applied game theory (a mathematical model) to understand and predict the behavior of the different component interactors in a fluorescent sensor for Hg^2+^ (Nie et al., 2018). In this paradigm, the aptamer was the “bait” molecule which was able to interact with two molecular players: mercury (Hg^2+^) and cobalt oxyhydroxide nanosheets (CoOOH). Depending on how the bait interacted with the players, either a turn-on (Hg^2+^) or a turn-off (Hg^2+^ and CoOOH) signal was produced. The multi-player turn-off sensor demonstrated reliable detection within the range of 20–600 nM, and exhibited a detection limit of 7.94 nM. Additionally, recovery of spiked Hg^2+^ in pond water was demonstrated with recoveries of 108.29 and 104.93% when 84.00 and 155.00 nM of Hg^2+^ was added to pond water, respectively.

### Multiplex Detection of Metal Ions

Whereas, many of the sensors described for the detection of heavy metal focus on one particular analyte, many researchers also report multiplex detection of multiple heavy metal ions. The described sensors ranged in complexity from simple fluorescent systems which rely on the formation of a metal dependent non-canonical base pair (either T-Hg^2+^-T or C-Ag^+^-C) to nanoparticle and electrochemical based methods (Zhang et al., 2018c; Abu-Ali et al., 2019; Berlina et al., 2019). A sensor which made use of metal stabilized base-pairing boasted detection limits of 5 × 10^−8^ and 9.3 × 10^−10^ mol/L for Hg^2+^ and Ag^+^, respectively (Zhang et al., 2018c). Further, recoveries of both silver and mercury ions were demonstrated in spiked tap water samples. Likewise, another metal-DNA interaction was exploited to demonstrate dual ion detection. In this case, G-Pb^2+^-G and T-Hg^2+^-T were strategically utilized to specifically aggregate AuNPs, resulting in a distinct color change of the solution in the presence of the target metal ion (Berlina et al., 2019). This assay, which could be performed in 20 min, was used to detect as little as 120 ng/mL of lead and 500 ng/mL of mercury. Furthermore, the qualitative colorimetric detection of lead and mercury in spiked lake water was demonstrated. Finally, Abu-Ali et al. also demonstrated the dual detection of Hg^2+^ and Pb^2+^ in environmental water samples by employing an electrochemical biosensor (Abu-Ali et al., 2019). In this design, aptamers were terminally labeled with a thiol (for attachment to the gold surface of the electrode) and a redox label (either ferrocene or methylene blue). Evaluation by cyclic voltammetry and impedance spectroscopy revealed detection limits as low as 0.1 ng/mL, and detection of metal ions in environmental water samples was demonstrated.

### Aquatic Toxins

Aquatic toxins, either exogenous or naturally occurring, can have devastating effects on wildlife, human health, and entire ecosystems. Table 1 highlights some recent examples of aptamer-based biosensors for the detection of aquatic toxins.

**Table 1:** Aptamer-based biosensors for the detection of aquatic toxins.

**Toxin**	**Sensor type**	**LDR/LOD**	**Detection matrix**	**References**
MC-LR	Photoelectrochemical detection based on core-shell CuS-TiO_2_ composite	LDR: 5.0 x 10^−5^ nM−250 nM LOD: 2.0 x 10^−5^ nM	Lake, river, and tap water	Tang et al., 2018a
MC-LR	Fluorescent detection based on DNA-templated copper nanoclusters	LDR: 0.005–1,200 μg/L LOD: 0.003 ng/L	Lake and drinking water	Zhang Y. et al., 2019
MC-LR	Nanomechanical sensing based on microcantilever array	LDR: 1–50 μg/L LOD: 1 μg/L	Tap water	Zhang et al., 2018a
NOD-R	Optical detection based on biolayer interferometry	LDR: 40–200 nM LOD: 167 pM	Tap water	Ouyang et al., 2018
MG	Turn-ON fluorescence detection	LDR: 0.05–2 μM LOD: 47.7 nM	Fish	Luo et al., 2019
SAX	Optical detection using SERS	LDR: 10–200 nM LOD: 11.7 nM	Shellfish	Cheng et al., 2019

*MC-LR, microcystin-LR; NOD-R, nodularin-R; MG, malachite green; SAX, saxitoxin; SERS, surface enhanced raman spectroscopy; LDR, Linear Dynamic Range; LOD, Limit of Detection*.

These toxins remain a persistent problem, as ecological instability and climate change can promote their existence, therefore governing agencies have set regulations on the most common and most problematic aquatic toxins. Aptamer-based sensors have demonstrated potential in detecting these toxins in environmental samples well below the regulatory limits, however there remains the challenge of translating these biosensors to portable, easy-to-use detection devices. With additional characterization and industrial collaboration, these parameters could be easily realized. Many of these examples require minimal sample preparation and the use of signaling methods (optical and chip-based detection) that can be incorporated into commercially available hand-held devices.

### Pesticides and Insecticides

Though the dangers of once liberally applied pesticides and insecticides have come to light, and governing agencies have set strict recommendations for use, the contamination of environmental sources by pesticides and their byproducts remains a significant challenge. Not only do pesticides have an effect on the local environment in which they are used, acute exposure and bioaccumulation of pesticides has also been shown to negatively impact humans as well. For these reasons, the development of rapid, and portable sensing devices for the detection of pesticides in environmental sources is required. Aptamer-based biosensors for the detection of pesticides and their byproducts have been reviewed (Yan X. et al., 2018; Zou et al., 2019). Recently, several colorimetric and fluorescent biosensors for the detection of pesticides have been described. The colorimetric methods are used to detect malathion, acetamiprid, chlorpyrifos, and isocarbophos residues and can be further subdivided into nanoparticle- and enzymatic-based methods.

Using indirect aptamer-controlled aggregation of silver nanoparticles, malathion was detected with a limit of 0.5 pM, and demonstrated recoveries ranging from 89 to 120% were observed from spiked lake water, tap water, and apple samples (Bala et al., 2018). In this work, the negatively charged unmodified aptamer interacted with a positively charged cationic peptide. When malathion was not present, the negatively charged nanoparticles repelled each other, and the solution was yellow. In the presence of malathion, the positively charged cationic peptide neutralized the nanoparticles, which then agglomerated resulting in a solution color of orange. Qi et al. took advantage of the ability of aptamers to directly control aggregation of gold nanoparticles for the detection of acetamiprid (Qi et al., 2020). In this case, the aptamer originally selected by He et al. (2011), adsorbed onto the surface of the AuNP in the absence of acetamiprid, leading to a distinct color change from red to blue. Rapid detection of acetamiprid in the range of 8.7–920 nM, with a visual detection limit of 75 nM (0.56 nM by UV Vis spectroscopy) was achieved. Furthermore, recoveries of spiked wastewater, soil, and cucumber samples ranged from 95.2 to 104.0%.

Employing the enzymatic approach, Xiang et al. incorporated an aptamer into a modified ELISA (enzyme-linked immunosorbent assay) to detect isocarbophos, yielding a detection limit of 0.05 μg/L and a linear concentration range of 0.05–23.142 μg/L (Xiang et al., 2019). Environmental water samples were spiked with isocarbophos, and demonstrated recoveries ranged from 84 to 97%. Finally, enzyme-based colorimetric detection of chlorpyrifos was achieved through the use of a NanoZyme (Weerathunge et al., 2019). Briefly, tyrosine-capped silver nanoparticles have intrinsic peroxidase-mimicking activity, which was controlled by the interaction of a chlorpyrifos specific aptamer and the surface of the nanoparticle. In the absence of the target pesticide, no signal was generated. However, in the presence of chlorpyrifos, detection was achieved in the linear range of 35–210 ppm and a detection limit of 11.3 ppm was demonstrated. Additionally, observed recoveries of chlorpyrifos spiked river water samples ranged from 98.8 to 103.6%. An alternative approach involved the development of a fluorescent biosensor. Arvand et al. described the use of an NH_2_-C6-modified aptamer (Jokar et al., 2017) conjugated quantum dots and graphene oxide to generate a sensing system for the detection of diazinon which had a linear dynamic range of 1.05–205.83 nM and a LOD of 0.13 nM (Arvand and Mirroshandel, 2019). Moreover, the recovery of diazinon from spiked river water, apple, and cucumber samples was demonstrated between 91.0 and 102%.

### Industrial Byproducts

A common challenge for environmental monitoring is the detection of harmful industrial byproducts. Recently aptamer-based biosensors have been reported for the detection of industrial byproducts such as 2,3′,5,5′-tetrachlorobiphenyl (also known as PCB72) in river water (Liu S. et al., 2019), 2-hydroxyfluorene in lake water (Liang et al., 2018), and hydroxylated polychlorinated biphenyl (OH-PCB) in lake water (Yang et al., 2018). However, by far the most common topic of recent aptamer research in this field is the detection of bisphenol A (BPA), a chemical used in the manufacturing of plastics and resins (Lee et al., 2019). Though there has been some controversy over the health risks of exposure to BPA, its leaching into exposure sources such as food, water, and the environment remains a concern. A common theme amongst aptamer-based BPA biosensors is the use of AuNPs in the sensing design. Lee et al., reported sensitive and selective detection of BPA over similar compounds using a previously truncated aptamer (Lee et al., 2017) to control AuNPs aggregation (Lee et al., 2019). In the presence of BPA in solution ranging from 0.001 to 1,000 ng/mL, a distinct color change from red to blue was observed, with a LOD of 1 pg/mL. Detection of BPA in rice samples which had been spiked was also demonstrated. Song and colleagues used a different approach in which the negatively charged aptamer, which was the full length version of that used by Lee et al. (2019), was able to interact with positively charged AuNPs [(+)AuNPs] (Jo et al., 2011; Qi et al., 2019). In the presence of BPA, the (+)AuNPs remained suspended in solution due to their repulsion, whereas without BPA the negatively charged aptamer forced the aggregation of the particles. This lead to a colorimetric signal which was dependent on the proximity of the (+)AuNPs, but further addition of luminol and AgNO_3_ to the reaction allows for a strong chemiluminescence signal in the presence of BPA and a weak signal in its absence. By this method, BPA was detected between the linear range of 0.1–40 ng/mL and with a LOD of 6.2 × 10^−2^ ng/mL. Interestingly, the sensor was used to detect BPA in soil obtained from an electronic waste dismantling site. The detected values were in good agreement with the values obtained by HPLC. Wang et al. reported a SERS-based biosensor that employs Au@Ag nanoparticles to generate a signal in the presence of BPA (Wang et al., 2018) based on the aptamer selected by Jo et al. (2011). In this assay, the negatively charged aptamer preferentially interacted with the BPA, such that in the presence of BPA the nanoparticles are distributed in solution (signal off) and in the absence of BPA the particles are aggregated resulting in an increased signal. This method, which was demonstrated to detect BPA in bottled water in 15 min, demonstrated a linear dynamic range of 0.01–1 ng/mL and a LOD of 2.8 pg/mL. Finally, Xu et al. used silver-coated gold nanostars (AgNS), which interacted with glucose oxidase modified BPA aptamer (Jo et al., 2011) to generate a SERS signal in the presence of BPA (Xu et al., 2018). Briefly, the BPA aptamer modified with glucose oxidase was incubated with the AuNS. In the absence of BPA, the aptamer conjugate is immobilized on the AuNS, and in the presence of the necessary reagents a signal is generated. Conversely, in the absence of BPA, the aptamer conjugate binds preferentially with BPA rather than the AuNS, reducing the generated signal. By this method, a linear dynamic range of 10^−16^-10^−12^ g/mL and a limit of detection of 5 × 10^−17^ g/mL were observed. Recoveries of BPA which had been spiked into tap water were demonstrated between 93.8 and 103.1%. One recent alternative approach utilized a peroxidase-mimic DNAzyme to generate a BPA-dependent signal (Xu et al., 2019). In this design, two complementary DNA probes, one containing the BPA aptamer (Jo et al., 2011) and part of the peroxidase mimic DNAzyme (probe A), and the other containing the remainder of the peroxidase mimic DNAzyme (probe B), interact to generate a chemiluminescence signal in the presence of H_2_O_2_ and luminol. When no BPA was present, the two probes hybridized to form an active DNAzyme leading to a chemiluminescent signal. When BPA was present, probe A preferentially interacted with the BPA, prevented the formation of the peroxidase mimic DNAzyme, and no chemiluminescent signal was generated. This method allowed for the detection of BPA with a reported limit of 5 nM.

### Pharmaceuticals

The availability and overuse of pharmaceutical and medicinal compounds has led to the contamination of environmental water sources. To understand the risks associated with second-hand exposure to these compounds, it is necessary to develop highly sensitive and specific biosensors for their detection in environmental sources. In particular, commonly prescribed medications like the chemotherapeutic agent doxorubicin (Bahner et al., 2018), common pain reliever ibuprofen (Ping et al., 2018), hormonal birth controls and hormone replacement therapies, and antibiotics have been the focus of recent biosensor efforts. In fact, a simple smartphone based portable colorimetric biosensor for the detection of ibuprofen was described which could detect the molecule in tap water and river water at spiked concentrations in the low ng/mL range (Ping et al., 2018). In this assay, the colorimetric signal generation depended on the interaction of the aptamers (both selected by Kim et al., 2010 and were reported to have micromolar K_d_s), AuNPs, and the target molecule (S- or R-ibuprofen) in a mechanism that was similar to the assay described in Figure 6. To perform a test, the user simply needed to set up the assay and calibrate the system, and then the smartphone-based program calculates the amount of red, green, and blue color in the sample tubes. The calibration data is then used to analyze each sample, and report a concentration to the user. Advantageously, this system could be easily adaptable to other aptamer-AuNP based sensing systems, it can be used for on-site detection, and the test results can be analyzed in less than 10 min.

Recent progress toward the detection of the hormone 17β-estradiol, a steroidal estrogen used in replacement therapy, is summarized in Table 2. Likewise, recent biosensors for the detection of antibiotics are summarized in Table 3.

**Table 2:** Aptamer-based biosensors for the detection of 17β-estradiol in environmental water sources.

**Sensor type**	**LDR and LOD**	**Detection matrix**	**References**
Fluorescent turn-ON sensor based on FRET	LDR: 0.1 ng/mL−10 μg/mL LOD: 0.1 ng/mL	Water, urine, and milk	Zhang et al., 2018b
SERS based detection using Au@Ag NP coated with 4-MBA	LDR: 0.1 pM−10 nM LOD: 0.05 pM	Wastewater and river water	Liu et al., 2018
Label-free electrochemical detection based on graphene and Au electrode	LDR: 0.07–10 pM LOD: 50 fM	Tap water	Liu M. et al., 2019
FRET based detection based on F-APT and graphite NPs	LDR: 0–20 ng/mL LOD: 1.02 ng/mL	groundwater	Qi et al., 2018
Detection achieved using a Field-effect transistor	LOD: 3.47 × 10^−11^ M	Tap water	Li et al., 2019

*FRET, Förster Resonance Energy Transfer; SERS, Surface Enhanced Raman Spectroscopy; 4-MBA, 4-mercaptobenzoic acid; F-APT, fluorescently labeled aptamer; NPs, nanoparticles; LDR, linear dynamic range; LOD, limit of detection*.

**Table 3:** Aptamer-based biosensors for the detection of antibiotics in environmental water sources.

**Antibiotic**	**Sensor type**	**LDR and LOD**	**Detection matrix**	**References**
Tetracycline	PEC	LDR: 0.4–14,370 ng/L LOD: 0.2 ng/L	Water	Dong et al., 2019
Oxytetracycline	EC	LDR: 2 × 10^−4^-1.0 nM LOD: 35.0 fM	Wastewater, urine, and milk	Zhou et al., 2019
Oxytetracycline	EC	LDR: 20–325 nM LOD: 2.5 nM	Wastewater, and tap water	Yildirim-Tirgil et al., 2019
Chloramphenicol	EC	LDR: 1–1,000 ng/mL LOD: 1 ng/mL	River water and milk	Cao et al., 2018
Aminoglycosides	FL	LDR: 200 nM−200 μM LOD: 26 nM	Milk	Tang et al., 2018b
Kanamycin	SERS	LDR: 1–100 nM LOD: 0.75 nM	Drinking water, tap water, orange juice, and milk	Nguyen et al., 2019

*PEC, photoelectrochemical; EC, electrochemical; FL, fluorescent; SERS, surface-enhanced raman spectroscopy; LDR, linear dynamic range; LOD, limit of detection*.

### Multiplex Detection of Environmental Contaminants in Water

**Figure 7:**
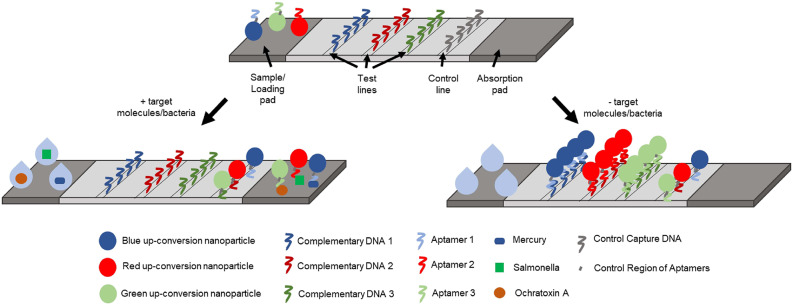
Schematic representation of the lateral-flow aptamer assay developed for the detection of multiple analytes in environmental water samples (Jin et al., 2018).

Up-conversion nanoparticles (UCNPs) are a unique class of optical nanoparticles that are often referred to as next generation fluorophores due to their ability to convert near-infrared excitation with lower energy into visible emissions using higher energy via a non-linear optical process (Wang M. et al., 2011). Recently, UCNPs have gained attention as signal reporters due to low background signals, unique luminescent properties, low signal-to-noise ratios, high resistance to photobleaching, and high sensitivity in both *in vivo* and *in vitro* applications. Using this principle, Jin et al. developed a lateral flow aptamer assay (LFAA: shown in Figure 7) for the simultaneous detection of multiple targets of interest using a smartphone-based portable device (Jin et al., 2018). In their design, three different amino-modified aptamer sequences exhibiting high specificity for mercury, ochratoxin A, and *Salmonella* were covalently bound to red, green, and blue UCNPs, respectively, to form aptamer-multicolored UCNP probes. As for the design of the LFAA, test lines were created by depositing streptavidin to act as an intermediate between the nitrocellulose paper and biotinylated complementary sequences to the aptamer to allow for the immobilization of the unbound probes and control sequences. All three probes were mixed with a sample solution containing mercury ions, ochratoxin A, and *Salmonella* for 30 min, then loaded onto the LFAA to allow for simultaneous detection of target analytes, as the probes were allowed to flow across the apparatus to reach the test zone. The test zones were washed and excited with a 980 nm laser allowing for the fluorescence of the UCNPs to be captured with a camera. Using this sensing platform, Jin et al. were able to achieve detection limits of 5 ppb, 3 ng/mL and 85 CFU/mL for mercury ions, ochratoxin A, and *Salmonella*, respectively. Multiplex detection in tap water was also demonstrated in less than 30 min. This sensitive, rapid, and low-cost device has immense potential to serve as a useful tool that has widespread applications in the field of food safety, environmental monitoring, and medical diagnostics.

## Aptamers for Monitoring Soil Quality

The detection of targets by aptamer-based biosensors which related to soil quality mainly fall under the categories of either heavy metals, or bacteria and bacterial toxins. The following sections describe examples of biosensors for the detection of environmental contaminants in soil.

### Monitoring Lead in Soil Using Aptamer-Based Biosensors

In an effort to address these challenges, Ding et al., described two aptamer-based sensors for the detection of lead (II) in soil samples (Ding et al., 2018, 2019). The most recent sensor takes advantage of the interaction between a G-rich lead binding aptamer, AuNPs, and polypyrrole screen printed onto an electrode to generate an electrochemical signal. This biosensor displayed a linear dynamic range of 0.5–25 ppb, a detection limit of 0.6 ppb, and its selectivity against common interfering ions was demonstrated. A year prior, Ding et al. reported a similar electrochemical biosensor which had a linear dynamic range of 0.5–10 nM, and a detection limit of 0.36 nM (Ding et al., 2018). Further, both methods demonstrated excellent selectivity against common interfering ions, and were used to detect lead (II) in soil obtained from a nearby farm with an accuracy that was not significantly different from the control measurements.

### Detection of Agricultural Toxins Present in Soil

The majority of research which focuses on the use of aptamers for the detection of agricultural toxins focuses on the detection of mycotoxins (summarized in Table 4). Mycotoxins, including but not limited to aflatoxins and ochratoxin, can cause severe health problems upon exposure. In fact, the International Agency for Research on Cancer has classified mycotoxins as possible human carcinogens (Jo et al., 2018). The rapid, sensitive, and portable detection of mycotoxins is of interest in multiple points along the food production pathway, from the farmers to retailers to consumers. Currently, traditional instrumentation (chromatography and mass spectrometry) as well as immunoassay-based detection allow for rapid and sensitive detection of mycotoxins, but still present limitations on use by non-experts in field settings. The sensors highlighted in Table 4 are not an exhaustive list of recently published mycotoxin binding aptamer-based biosensors, however, they are examples of relatively simple assays which could be conducted in a reasonable period of time for potential on-site analysis and environmental monitoring of aqueous and soil samples. In particular, the detection of aflatoxin B1 in less than 1 min was demonstrated in soil, using a one-pot mix-to-read assay (Zhao Z. et al., 2019).

**Table 4:** Aptamer-based biosensors for the detection of agricultural toxins.

**Toxin**	**Sensor type**	**LDR/LOD**	**Detection matrix**	**References**
AFB1	Fluorescent detection using a capillary array	LDR: 0.5 nM−1.0 μM LOD: 0.5 nM	Wine and beer	Sun and Zhao, 2018
AFB1	Electrochemical detection using graphene oxide and gold nanowires	LDR: 5.0–750.0 pM LDR: 1.4 pM	Pistachios	Nodoushan et al., 2019
AFB1	Fluorescent detection based on aggregation-induced emission dyes	LDR: 0–500 ng/mL LOD: 0.29 ng/mL	Food, soil, and tap water	Zhao Z. et al., 2019
OTA	Fluorescent detection based on FRET	LDR: 5–700 ng/mL LOD: 0.38 ng/mL	Coffee and oats	Zeng et al., 2019
OTA	Electrochemical detection using a metal organic framework as signal probe	LDR: 0.05–100 ng/mL LOD: 10 pg/mL	Wine	Li D. L. et al., 2018
OTA	Luminescent detection based on LRET	LDR: 0.1–1,000 ng/mL LOD: 0.098 ng/mL	Wine, grape juice, and beer	Jo et al., 2018

*AFB1, aflatoxin B1; OTA, ochratoxin A; FRET, Förster Resonance Energy Transfer; LRET, Luminescence Resonance Energy Transfer; LDR, Linear Dynamic Range; LOD, Limit of Detection*.

## Aptamers for Monitoring Air Quality

One area of aptamer-based environmental monitoring that could use more researchers' attention is air quality. With the exception of some work that focused on developing optical [fluorescent (Ammanath et al., 2018; Wei W. et al., 2018), colorimetric (Ammanath et al., 2018; Choodet et al., 2019; Toomjeen et al., 2019), and resonance light scattering (Tao et al., 2018)] and electrochemical (Zheng et al., 2019) biosensors for 8-hydroxy-2′-deoxyguanosine (8-OHdG), a biomarker for oxidative stress that can be detected in urine and has been associated with air pollution (Tao et al., 2018), only one recent study has focused on using aptamers to monitor air quality.

**Figure 8:**
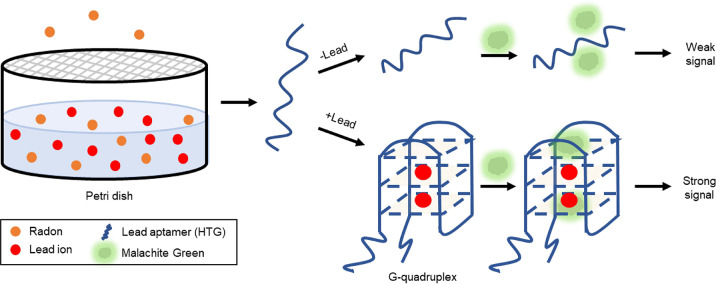
Indirect detection of accumulated radon via aptamer-based lead ion detection (Li S. et al., 2018).

Radon is a colorless, odorless, toxic gas which has been designated by the World Health Organization and the International Cancer Organization as a first-class environmental carcinogen (Li S. et al., 2018). In fact, exposure to radon, which exists naturally in rocks and soil, water sources, and as a combustion product of coal and natural gas, is the second leading risk factor for developing lung cancer (Li S. et al., 2018). Currently challenges exist in both the detection of radon at low doses, and measuring accumulation as existing methods are expensive and potentially dangerous to human health. Therefore, it is necessary to develop new technologies which can detect radon. Li et al. developed a label-free fluorescent biosensor, represented schematically in Figure 8, which exploits the radioactive decay of ^222^Rn to Pb^2+^ in order to detect radon (Li S. et al., 2018).

In the design of this fluorescent biosensor, a petri dish containing 10 mL of 0.2% acetic acid which absorbed the decayed lead ion was capped with a mixed cellulose microporous membrane to prevent airborne lead contamination. The collection dish was placed in a radon chamber at room temperature for time intervals ranging from 2 to 84 h, chosen to cover the half-life of radon. A small aliquot of the acetic acid solution was added to a reaction containing buffer and lead aptamer (HTG). Following a 1.5 h incubation at 37°C, malachite green (MG) was added, and following a subsequent 10 min incubation period, the fluorescence of the solution was measured. In principle, when no lead ion was present, the aptamer remained largely unstructured and minimally interacted with the MG producing only a weak fluorescent signal. In the presence of lead ion, the G-rich aptamer (T30695) underwent a structural transition to a G-quadruplex (Khoshbin et al., 2019). Interestingly, the G-rich sequence used in the study showed a higher affinity to lead than the peroxidase mimic DNAzyme (PS2.M) used in the water assays, a consideration for future work. Due to the physical orientation, the malachite green interacted with the G-tetrads formed after the aptamer bound to lead, via π-stacking and produced strong fluorescence. By this method, the concentration of lead ion detected was directly proportionate to the amount of gaseous radon measured by traditional methods. The biosensor displayed a linear dynamic range of 6.87 × 10^3^ to 3.49 × 10^5^ Bq·h/m^3^ and a LOD of 2.06 × 10^3^ Bq·h/m^3^. Importantly, the sensitivity of the device to lead over other common metal ions was demonstrated. These parameters compare favorably to those reported for typical methods for the detection of both radon, and lead ion. Additionally, there was no statistical difference between the measurements obtained using a commercially available radon monitor (RAD7) and the described biosensor.

Despite the challenges of selecting aptamers for targets in the gas phase, precedence suggests it may still be possible to take advantage of the programmability of functional nucleic acid affinity probes to build biosensors for the indirect detection of toxic gases. As an example of this approach, one study described the detection of H_2_S using a copper ion-dependent DNAzyme to generate a fluorescent signal (Yue et al., 2019), and another study used a peroxidase mimic DNAzyme to generate a colorimetric signal (Kang et al., 2017). The limits of detection of H_2_S for these biosensors were 200 and 410 nM respectively. Further, recoveries from spiked aqueous solutions ranging from 91.0 to 108.0% were demonstrated.

Other common toxic gases which may be interesting targets for the development of aptamer-based biosensors include: nitrogen dioxide, sulfur dioxide, and hydrogen sulfide (Khan et al., 2019). The detection of these gases, which can occur naturally and as the byproduct of some industrial processes, is absolutely essential to air quality monitoring. Acute and long-term exposure to these gases can drastically affect the quality of human life, wildlife, vegetation, and infrastructure (Khan et al., 2019). Currently, electrochemical methods are commonly used to monitor ambient gas levels (Khan et al., 2019). Given the prevalent reporting of aptamer-based electrochemical sensors, the most logical incorporation of aptamers for gas sensing may fall in this realm.

## Incorporation of Aptamers Into Wearable and Sentinel Devices

**Figure 9:**
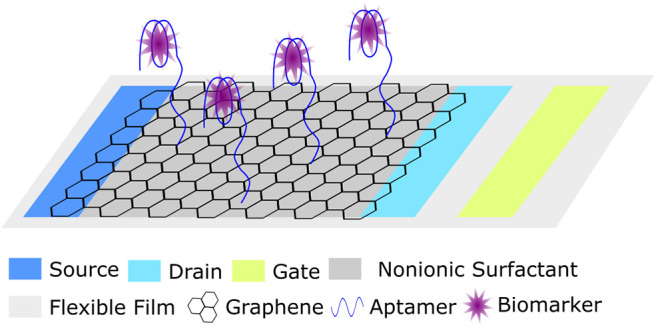
Schematic representation of a wearable ultra-flexible graphene field effect transistor (GFET) for biomarker detection (Wang et al., 2019).

The incorporation of functional nucleic acids, including aptamers into wearable and sentinel nanomaterial devices has seen the most progress in the medical realm. Several nanomaterial constructed wearable sensors have been recently reviewed (Yao et al., 2018; Kalambate et al., 2019). In particular, researchers have made progress in developing wearable sensors by using nanomaterials such as carbon nanotubes and nanotechnologies such as field-effect transistors to measure body temperature, monitor electrophysiology, measure mechanical strain, sense pressure and touch, detect biomarkers, and monitor environmental exposure. An opportunity exists for aptamers to be incorporated into many of these devices to improve sensitivity and biocompatibility, as well as decrease production costs. Illustrating the feasibility of this, Wang et al. recently described an ultra-flexible graphene-based field effect transistor (GFET) that employs an aptamer as the molecular recognition unit for the picomolar detection of an inflammatory cytokine biomarker (Wang et al., 2019). The device (Figure 9), which is made up of aptamer-modified graphene conducting channel on an ultra-flexible thin film of Mylar, was successfully incorporated into a skin patch as well as a contact lens. Adaptations of this work could be widely applied in environmental monitoring. For example, these devices may prove useful in monitoring human exposure to dangerous chemicals in particular environments.

**Figure 10:**
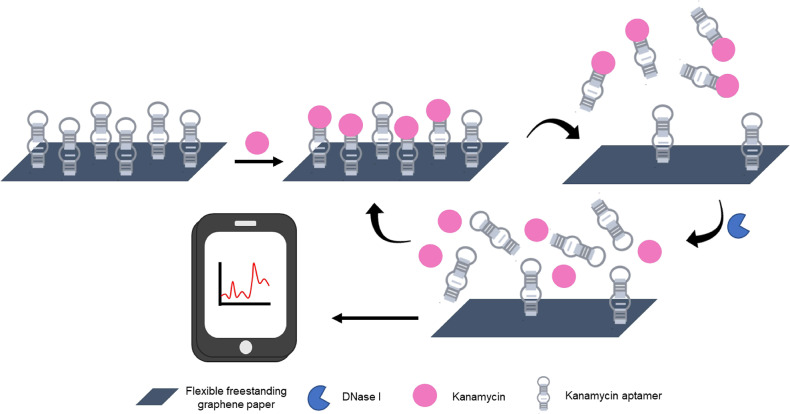
Schematic diagram of the sensing principle behind aptamer-based kanamycin detection using freestanding graphene paper (GNP) and nuclease-assisted amplification strategy (Yao et al., 2019).

Another example where a developed wearable device could be easily adapted for environmental monitoring was described by Yao et al. who were interested in developing a freestanding graphene paper-based wireless device for the detection of the antibiotic kanamycin (Yao et al., 2019). The ultrasensitive and rapid detection of antibiotics by aptamer-based devices has been demonstrated multiple times (as described in previous sections). In this example, shown schematically in Figure 10, the flexible graphene-paper device which combined aptamer-based detection and nuclease assisted signal amplification was incorporated into a sensing platform that allowed for fg/mL detection of kanamycin using a wireless transmitting detector and a smart phone.

**Figure 11:**
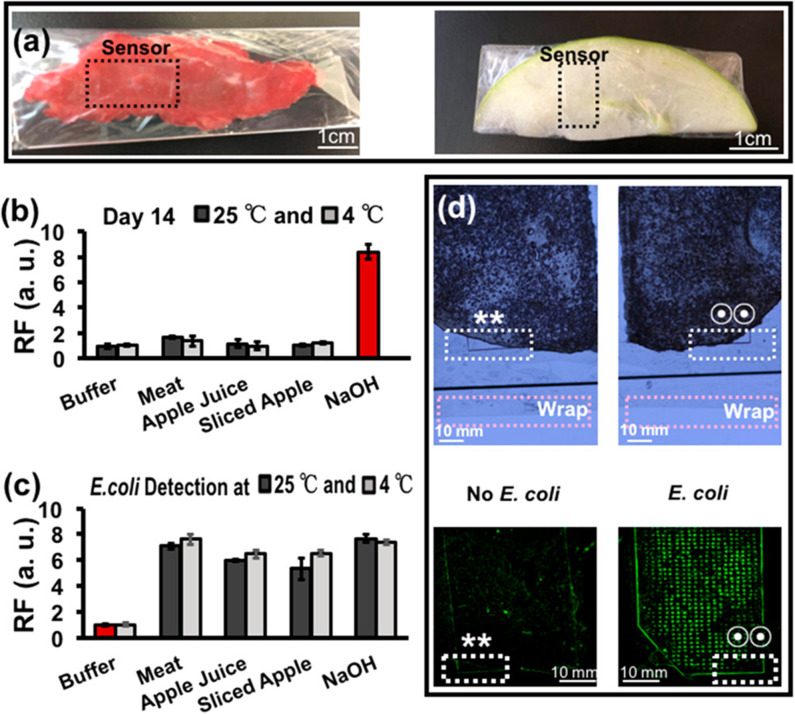
Detection of bacteria in food by RNA-cleaving DNAzyme (RFD-EC1) reactive sentinel wraps. **(a)** Meat and apple samples were wrapped in the RFD-EC1 incorporated polymer wraps. The RFD-EC1 were incubated in buffer, meat, apple juice sliced apple or NaOH either without **(b)** or with **(c)** bacteria present for 14 days. A clear fluorescence signal was generated by RFD-EC1 in the presence of *E. coli* **(d)**. Likewise, the RFD-EC1 incorporated food wrap revealed distinct fluorescence when the food samples had been inoculated with a bacteria mixture with and without *E. coli*. Overlapping regions in the bright field (top) and fluorescence (bottom) are represented by ** (no *E. coli*) and two-circle symbol (*E. coli*). Reprinted (adapted) with permission from Yousefi et al. (2018). Copyright (2018) American Chemical Society. Permission has been obtained from American Chemical Society.

As a final example, the development of functional nucleic acid-based sentinel wraps for the real-time monitoring of food contamination was described by Yousefi et al. (2018). In this work an RNA-cleaving DNAzyme, a functional nucleic acid that is similar to an aptamer but has the added functionality of catalytic self-cleavage, was incorporated as a microarray into cyclo-olefin polymer film. In the presence of *E. coli* in the meat sample, the film displayed a very distinct fluorescent signal (shown in Figure 11).

These examples demonstrate the feasibility of incorporating functional nucleic acids into wearable and sentinel devices. It stands to reason then, that if researchers are capable of designing sensors for human skin, sensors could surely be designed for the skin of fish, birds, or other mammals for the purposes of monitoring bioaccumulation and environmental contaminant exposure. Likewise, researchers could focus on using aptamers to develop wearable devices for monitoring plant life. Giraldo et al. have recently reviewed progress toward the development of “smart” plant sensors, which, when triggered by internal and environmental stimuli, could provide digital information to electronic devices and systems for autonomous environmental monitoring (Giraldo et al., 2019). For example, wearable plant sensors which monitor basic crop health were discussed, as well as preliminary examples which were presented where two-way communication between smart plant sensors and machines allow plants to control their own environment, with the eventual purpose of this plant-based feedback assisting with automated crop management.

## Perspectives and Recommendations

The field of environmental monitoring has surely benefited from the development of aptamer and aptamer-nanomaterial based biosensors for the detection of relevant contaminant analytes. In the past few years, there have been several advances in the development of portable aptamer-based devices which provide rapid, on-site single and multiplex detection of an array of target analytes. Despite this progress, challenges still remain in the field in translating bench science to on-site detection. To bridge the gap, researchers should focus on ensuring their designs adhere to the ASSURED criteria described by the WHO.

Oftentimes the presented research stops at the demonstration of biosensor detection in spiked environmental samples. To translate much of this work to practical use, it is necessary to evaluate the function of these biosensors with minimal sample preparation, to validate detection of naturally occurring contaminants in complex environmental samples, and to demonstrate reliable, user-friendly, on-site detection of target analytes. Though the detection matrix relevant to most environmental monitoring, water, is relatively non-complex (compared to, for example, biological samples like blood), there still exists unique challenges for the on-site detection of contaminant analytes. Where a hospital or medical lab may offer access to conveniences like electricity and refrigeration, environmental samples are collected and stored in vastly different internal and external climates which can change rapidly and affect the quality of downstream processing.

As evidenced by the discussed examples, researchers interested in aptamer-based environmental monitoring should draw inspiration from the intersectionality of nanomaterials and other nucleic acid-based approaches being used for flora and fauna monitoring, with translatable progress in other fields such as point-of-care devices and personalized medicine. Progress will surely come from combing these, and other innovative approaches, to provide end-users with inexpensive, reliable, biosensors for the detection of environmental contaminants.

## Author Contributions

The review was researched and prepared by EM, JN, and YL. Figures and Tables were prepared by EM and JN with guidance from YL.

## Conflict of Interest

The authors declare that the research was conducted in the absence of any commercial or financial relationships that could be construed as a potential conflict of interest.
